# Bis(3-nitro­phen­yl) sulfone

**DOI:** 10.1107/S160053680801917X

**Published:** 2008-07-05

**Authors:** Wei Yao, Fang-Shi Li, Da-Sheng Yu, Mei-Juan Liu, Jin-Na Zhu

**Affiliations:** a Department of Applied Chemistry, College of Science, Nanjing University of Technology, Xinmofan Road No. 5, Nanjing 210009, People’s Republic of China

## Abstract

The asymmetric unit of the title compound, C_12_H_8_N_2_O_6_S, an important diphenyl sulfone derivative, contains one half-mol­ecule; a mirror plane passes through the SO_2_ group. The dihedral angle between the two symmetry-related benzene rings is 40.10 (13)°. An intra­molecular C—H⋯O hydrogen bond results in the formation of a five-membered ring, which adopts an envelope conformation.

## Related literature

For related literature, see: Ayyangar *et al.* (1981 ▶); Amer *et al.* (1989 ▶). For bond-length data, see: Allen *et al.* (1987 ▶).
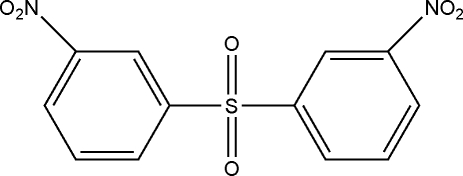


## Experimental

### 

#### Crystal data

C_12_H_8_N_2_O_6_S
*M* *_r_* = 308.27
Orthorhombic, 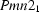

*a* = 20.260 (4) Å
*b* = 5.9380 (12) Å
*c* = 5.3770 (11) Å
*V* = 646.9 (2) Å^3^
*Z* = 2
Mo *K*α radiation
μ = 0.28 mm^−1^
*T* = 294 (2) K
0.30 × 0.20 × 0.10 mm

#### Data collection

Enraf–Nonius CAD-4 diffractometer
Absorption correction: ψ scan (North *et al.*, 1968 ▶) *T*_min_ = 0.920, *T*_max_ = 0.972
1304 measured reflections
674 independent reflections
624 reflections with *I* > 2σ(*I*)
*R*_int_ = 0.028
3 standard reflections frequency: 120 min intensity decay: none

#### Refinement

*R*[*F* ^2^ > 2σ(*F* ^2^)] = 0.032
*wR*(*F* ^2^) = 0.086
*S* = 1.00
674 reflections
101 parameters
H-atom parameters constrained
Δρ_max_ = 0.25 e Å^−3^
Δρ_min_ = −0.22 e Å^−3^
Absolute structure: Flack (1983 ▶), with no Friedel pairs
Flack parameter: −0.11 (15)

### 

Data collection: *CAD-4 Software* (Enraf–Nonius, 1989 ▶); cell refinement: *CAD-4 Software*; data reduction: *XCAD4* (Harms & Wocadlo, 1995 ▶); program(s) used to solve structure: *SHELXS97* (Sheldrick, 2008 ▶); program(s) used to refine structure: *SHELXL97* (Sheldrick, 2008 ▶); molecular graphics: *ORTEP-3 for Windows* (Farrugia, 1997 ▶); software used to prepare material for publication: *SHELXL97*.

## Supplementary Material

Crystal structure: contains datablocks I, global. DOI: 10.1107/S160053680801917X/hk2478sup1.cif

Structure factors: contains datablocks I. DOI: 10.1107/S160053680801917X/hk2478Isup2.hkl

Additional supplementary materials:  crystallographic information; 3D view; checkCIF report

## Figures and Tables

**Table 1:** Hydrogen-bond geometry (Å, °)

*D*—H⋯*A*	*D*—H	H⋯*A*	*D*⋯*A*	*D*—H⋯*A*
C4—H4*A*⋯O1	0.93	2.58	2.928 (4)	102

**Fig. 1.**
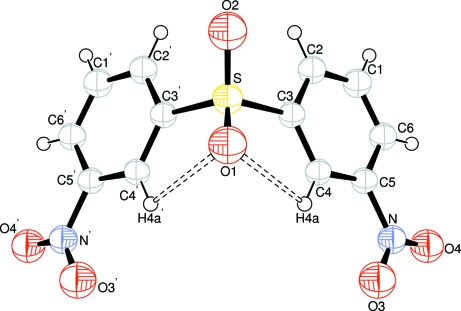
The molecular structure of the title molecule, with the atom-numbering scheme [symmetry code: (') -x, y, z]. Hydrogen bonds are shown as dashed lines.

## supplementary crystallographic information

### Comment

The title compound, (I), is used for preparing 3,3'-diaminodiphenyl sulfone (Ayyangar *et al.*,  1981). As part of our studies in this area, we report herein the synthesis and crystal structure of (I).

The asymmetric unit of (I) (Fig. 1) contains one half molecule. The bond lengths (Allen *et al.*,  1987) and angles are within normal ranges. The dihedral angle between the two symmetry related bezene rings is 139.90 (13)°. The intramolecular C-H···O hydrogen bond (Table 1) results in the formation of a five-membered non-planar ring: (S/O1/C3/C4/H4A), in which it adopts envelope conformation, with O1 atom displaced by -0.494 (3) Å from the planes of the other ring atoms.

### Experimental

The title compound, (I), was prepared according to the literature method (Amer *et al.*,  1989). Crystals suitable for X-ray analysis were obtained by dissolving (I) (0.2 g) in dichloroethane (25 ml) and evaporating the solvent slowly at room temperature for about 7 d.

### Refinement

H atoms were positioned geometrically, with C-H= 0.93 Å for aromatic H, and constrained to ride on their parent atoms, with U_iso_(H) = 1.2U_eq_(C).

### Crystal data

**Table:**

C_12_H_8_N_2_O_6_S	*F*_000_ = 316
*M**_r_* = 308.27	*D*_x_ = 1.583 Mg m^−^^3^
Orthorhombic, *P**m**n*2_1_	Mo *K*α radiation λ = 0.71073 Å
Hall symbol: P 2ac -2	Cell parameters from 25 reflections
*a* = 20.260 (4) Å	θ = 10–13º
*b* = 5.9380 (12) Å	µ = 0.28 mm^−^^1^
*c* = 5.3770 (11) Å	*T* = 294 (2) K
*V* = 646.9 (2) Å^3^	Block, light yellow
*Z* = 2	0.30 × 0.20 × 0.10 mm

### Data collection

**Table:**

Enraf–Nonius CAD-4 diffractometer	*R*_int_ = 0.028
Radiation source: fine-focus sealed tube	θ_max_ = 25.2º
Monochromator: graphite	θ_min_ = 2.0º
*T* = 294(2) K	*h* = −24→24
ω/2θ scans	*k* = −7→0
Absorption correction: ψ scan(North *et al.*, 1968)	*l* = 0→6
*T*_min_ = 0.920, *T*_max_ = 0.972	3 standard reflections
1304 measured reflections	every 120 min
674 independent reflections	intensity decay: none
624 reflections with *I* > 2σ(*I*)	

### Refinement

**Table:**

Refinement on *F*^2^	H-atom parameters constrained
Least-squares matrix: full	*w* = 1/[σ^2^(*F*_o_^2^) + (0.06*P*)^2^ + 0.078*P*] where *P* = (*F*_o_^2^ + 2*F*_c_^2^)/3
*R*[*F*^2^ > 2σ(*F*^2^)] = 0.032	(Δ/σ)_max_ < 0.001
*wR*(*F*^2^) = 0.086	Δρ_max_ = 0.25 e Å^−^^3^
*S* = 1.00	Δρ_min_ = −0.22 e Å^−^^3^
674 reflections	Extinction correction: SHELXL97 (Sheldrick, 2008), Fc^*^=kFc[1+0.001xFc^2^λ^3^/sin(2θ)]^-1/4^
101 parameters	Extinction coefficient: 0.069 (8)
Primary atom site location: structure-invariant direct methods	Absolute structure: Flack (1983), with no Friedel pairs
Secondary atom site location: difference Fourier map	Flack parameter: −0.11 (15)
Hydrogen site location: inferred from neighbouring sites	

### Special details

**Table:**

Geometry. All e.s.d.'s (except the e.s.d. in the dihedral angle between two l.s. planes) are estimated using the full covariance matrix. The cell e.s.d.'s are taken into account individually in the estimation of e.s.d.'s in distances, angles and torsion angles; correlations between e.s.d.'s in cell parameters are only used when they are defined by crystal symmetry. An approximate (isotropic) treatment of cell e.s.d.'s is used for estimating e.s.d.'s involving l.s. planes.
Refinement. Refinement of F^2^ against ALL reflections. The weighted R-factor wR and goodness of fit S are based on F^2^, conventional R-factors R are based on F, with F set to zero for negative F^2^. The threshold expression of F^2^ > 2sigma(F^2^) is used only for calculating R-factors(gt) etc. and is not relevant to the choice of reflections for refinement. R-factors based on F^2^ are statistically about twice as large as those based on F, and R- factors based on ALL data will be even larger.

### Fractional atomic coordinates and isotropic or equivalent isotropic displacement parameters (Å^2^)

**Table:**

	*x*	*y*	*z*	*U*_iso_*/*U*_eq_	
S	0.5000	0.08254 (19)	0.9607 (2)	0.0438 (4)	
N	0.68253 (13)	0.0542 (4)	0.2895 (5)	0.0422 (6)	
O1	0.5000	−0.1563 (5)	0.9183 (9)	0.0626 (12)	
O2	0.5000	0.1705 (7)	1.2095 (7)	0.0663 (11)	
O3	0.66204 (14)	−0.1343 (4)	0.2403 (5)	0.0660 (8)	
O4	0.72681 (13)	0.1460 (4)	0.1767 (5)	0.0599 (7)	
C1	0.64732 (14)	0.4956 (5)	0.7589 (8)	0.0451 (8)	
H1B	0.6638	0.6351	0.8072	0.054*	
C2	0.59364 (14)	0.4066 (5)	0.8822 (7)	0.0410 (7)	
H2B	0.5741	0.4842	1.0131	0.049*	
C3	0.56929 (13)	0.1978 (5)	0.8063 (5)	0.0347 (7)	
C4	0.59799 (14)	0.0791 (4)	0.6139 (6)	0.0341 (6)	
H4A	0.5816	−0.0603	0.5648	0.041*	
C5	0.65163 (13)	0.1743 (5)	0.4973 (6)	0.0356 (6)	
C6	0.67683 (14)	0.3828 (4)	0.5672 (7)	0.0410 (7)	
H6A	0.7130	0.4442	0.4852	0.049*	

### Atomic displacement parameters (Å^2^)

**Table:**

	*U*^11^	*U*^22^	*U*^33^	*U*^12^	*U*^13^	*U*^23^
S	0.0316 (5)	0.0543 (6)	0.0455 (7)	0.000	0.000	0.0169 (6)
N	0.0448 (13)	0.0450 (14)	0.0367 (14)	0.0071 (11)	0.0003 (12)	−0.0003 (11)
O1	0.0408 (17)	0.0472 (16)	0.100 (3)	0.000	0.000	0.032 (2)
O2	0.049 (2)	0.114 (3)	0.0364 (18)	0.000	0.000	0.016 (2)
O3	0.0784 (19)	0.0580 (15)	0.0615 (18)	−0.0025 (12)	0.0081 (16)	−0.0211 (14)
O4	0.0545 (14)	0.0738 (16)	0.0512 (15)	0.0009 (12)	0.0190 (12)	0.0005 (14)
C1	0.0374 (14)	0.0341 (14)	0.064 (2)	−0.0016 (12)	−0.0022 (16)	−0.0079 (16)
C2	0.0370 (15)	0.0400 (15)	0.0460 (17)	0.0078 (12)	−0.0021 (14)	−0.0055 (14)
C3	0.0274 (12)	0.0381 (14)	0.0386 (16)	0.0013 (11)	−0.0031 (12)	0.0081 (13)
C4	0.0341 (13)	0.0323 (12)	0.0359 (16)	−0.0004 (11)	−0.0049 (13)	0.0041 (14)
C5	0.0326 (13)	0.0370 (13)	0.0373 (14)	0.0067 (10)	−0.0038 (13)	0.0026 (13)
C6	0.0353 (14)	0.0364 (14)	0.0511 (19)	−0.0024 (12)	0.0046 (15)	0.0033 (14)

### Geometric parameters (Å, °)

**Table:**

S—O2	1.436 (4)	C1—H1B	0.9300
S—O1	1.437 (3)	C2—C3	1.395 (4)
S—C3	1.769 (3)	C2—H2B	0.9300
S—C3^i^	1.769 (3)	C3—C4	1.380 (4)
N—O4	1.212 (4)	C4—C5	1.376 (4)
N—O3	1.223 (3)	C4—H4A	0.9300
N—C5	1.466 (4)	C5—C6	1.391 (4)
C1—C6	1.367 (5)	C6—H6A	0.9300
C1—C2	1.379 (4)		
			
O2—S—O1	120.5 (3)	C3—C2—H2B	120.7
O2—S—C3	107.24 (14)	C4—C3—C2	121.6 (3)
O1—S—C3	107.92 (15)	C4—C3—S	119.2 (2)
O2—S—C3^i^	107.24 (14)	C2—C3—S	119.2 (3)
O1—S—C3^i^	107.92 (15)	C5—C4—C3	117.7 (3)
C3—S—C3^i^	105.06 (18)	C5—C4—H4A	121.2
O4—N—O3	123.7 (3)	C3—C4—H4A	121.2
O4—N—C5	118.6 (3)	C4—C5—C6	122.2 (3)
O3—N—C5	117.7 (3)	C4—C5—N	119.0 (3)
C6—C1—C2	121.3 (3)	C6—C5—N	118.8 (3)
C6—C1—H1B	119.3	C1—C6—C5	118.6 (3)
C2—C1—H1B	119.3	C1—C6—H6A	120.7
C1—C2—C3	118.6 (3)	C5—C6—H6A	120.7
C1—C2—H2B	120.7		
			
C6—C1—C2—C3	−0.4 (5)	S—C3—C4—C5	179.7 (2)
C1—C2—C3—C4	0.6 (5)	C3—C4—C5—C6	−0.1 (4)
C1—C2—C3—S	−179.4 (2)	C3—C4—C5—N	−179.0 (2)
O2—S—C3—C4	152.5 (2)	O4—N—C5—C4	175.0 (3)
O1—S—C3—C4	21.3 (3)	O3—N—C5—C4	−5.2 (4)
C3^i^—S—C3—C4	−93.6 (2)	O4—N—C5—C6	−3.9 (4)
O2—S—C3—C2	−27.5 (3)	O3—N—C5—C6	175.9 (3)
O1—S—C3—C2	−158.6 (3)	C2—C1—C6—C5	0.0 (5)
C3^i^—S—C3—C2	86.4 (3)	C4—C5—C6—C1	0.3 (5)
C2—C3—C4—C5	−0.3 (4)	N—C5—C6—C1	179.2 (3)

Symmetry codes: (i) −*x*+1, *y*, *z*.

### Hydrogen-bond geometry (Å, °)

**Table:**

*D*—H···*A*	*D*—H	H···*A*	*D*···*A*	*D*—H···*A*
C4—H4A···O1	0.93	2.58	2.928 (4)	102
